# Central synthesis of temporomandibular dysfunction

**DOI:** 10.1080/07853890.2021.1896443

**Published:** 2021-09-28

**Authors:** Inês Gonçalves, Irina José, João Jerónimo, Maura Almeida, Paula Moleirinho Alves, Catarina Ramos, Ângela Maria Pereira

**Affiliations:** a Department of Physiotherapy, Escola Superior de Saúde Egas Moniz (ESSEM), Egas Moniz Cooperativa de Ensino Superior, Caparica, Portugal; b Centro de Investigação Interdisciplinar Egas Moniz (CiiEM), Egas Moniz Cooperativa de Ensino Superior, Caparica, Portugal; c Hospital Garcia de Orta, Almada, Portugal

## Abstract

**Introduction:**

Temporomandibular dysfunction (TMD) is a group of conditions that affect bone structures and soft tissues of the orofacial region and are characterised mainly by pain [1]. Patients with TMD often have chronic pain, which in turn results from the mechanisms of sensitisation which is thus responsible for the hypersensitivity of pain [2]. Central sensitisation can be examined experimentally using a conditioned pain modulation paradigm; it can function as a form of inhibition of pain in humans [3]. This study aim to evaluate the conditioned modulation of pain in patients with temporomandibular dysfunction and chronic pain, and also to relate the influence that it has on anxiety and quality of life.

**Materials and methods:**

An analytic observational study was carried out, involving a group of 19 individuals with chronic pain (34.1 ± 14.9 yrs), and sample selection was performed using Research Diagnostic Criteria for Temporomandibular Disorder. (RDC/TMD). The subjects were submitted to the application of a mechanical (algometer) and thermal stimulus (ice) alone and to two mechanical and thermal stimuli simultaneously and independently. The interval application between stimulus, isolated and simultaneously was 5 min. All participants signed informed consent. The study was approved by the Ethics Committee of the Egas Moniz.

**Results:**

It was verified that the intensity of the pain perceived by the patients in the orofacial region during the simultaneous application of the two mechanical stimuli was in 100% of the cases lower than that perceived during the application of one stimulus. Regarding the thermal stimuli, it was verified that the intensity of the pain perceived in the orofacial region during the simultaneous application of the two thermal stimuli was 47% of the times inferior to that perceived during the application of one stimulus.

**Discussion and conclusions:**

The decrease in pain prediction in the orofacial region when two simultaneous stimuli were applied is in agreement with the principles of conditioned pain modulation, which seems to indicate that individuals with TMD have central sensitisation [3]. Given the small size of the sample and the small number of studies carried out on the present theme, it is suggested to carry out new studies with larger samples in the future.
